# Growth Mechanism of Seed-Layer Free ZnSnO_3_ Nanowires: Effect of Physical Parameters

**DOI:** 10.3390/nano9071002

**Published:** 2019-07-11

**Authors:** Ana Rovisco, Rita Branquinho, Jorge Martins, Elvira Fortunato, Rodrigo Martins, Pedro Barquinha

**Affiliations:** i3N/CENIMAT, Department of Materials Science, Faculty of Science and Technology, Universidade NOVA de Lisboa and CEMOP/UNINOVA, Campus de Caparica, 2829-516 Caparica, Portugal

**Keywords:** nanowires, ZnSnO_3_, ZTO, hydrothermal synthesis, growth mechanism

## Abstract

ZnSnO_3_ semiconductor nanostructures have several applications as photocatalysis, gas sensors, and energy harvesting. However, due to its multicomponent nature, the synthesis is far more complex than its binary counter parts. The complexity increases even more when aiming for low-cost and low-temperature processes as in hydrothermal methods. Knowing in detail the influence of all the parameters involved in these processes is imperative, in order to properly control the synthesis to achieve the desired final product. Thus, this paper presents a study of the influence of the physical parameters involved in the hydrothermal synthesis of ZnSnO_3_ nanowires, namely volume, reaction time, and process temperature. Based on this study a growth mechanism for the complex Zn:Sn:O system is proposed. Two zinc precursors, zinc chloride and zinc acetate, were studied, showing that although the growth mechanism is inherent to the material itself, the chemical reactions for different conditions need to be considered.

## 1. Introduction

As a result of its impressive multifunctionality, ZnO-based thin films and nanostructures have received a lot of attention in the last 10 years [1,2,3,4,5]. While ZnO on its own captured a large interest, doping or mixing with other binary compounds brings a new level of possibilities, as the material properties can be improved/tailored to different applications depending on the cationic ratio. This has been widely explored in oxide thin films, with materials such as indium-gallium-zinc oxide (IGZO) [6,7,8] or with more sustainable approaches that avoid critical raw materials (In and Ga) [9], as zinc-tin oxide (ZTO) [10,11]. While in thin film form one of the major arguments of these multicomponent materials has been their amorphous structure, highly desirable for uniform large area electronics, when moving to nanostructure synthesis it is important to consider the different crystalline phases that are possible to achieve. ZTO structures crystalize by solid-state reaction in the stable inverse spinel ortho-stannate Zn_2_SnO_4_ phase [12,13]. Nonetheless, it can also crystalize in metastable ZnSnO_3_ phase, either in perovskite (orthorhombic, orth, or face centered, fcc) [14] or rhombohedral [15] forms. Both of these phases can result in nanostructures with different shapes and different properties, thus providing a large degree of multifunctionality with this material system [16,17,18,19,20]. Zn_2_SnO_4_ is an n-type semiconductor with high mobilities in the order of 112 cm^2^ V^−1^ s^−1^ and a reported band gap of 3.6 eV for nanostructures [21,22]. On the other hand, ZnSnO_3_ is reported as having a high polarization along the *z*-axis of ~59 μC/cm^2^, which is much higher than that of ZnO (~5 μC/cm^2^) [18,23,24], and also as a ferroelectric material [19,25]. Its band gap of 3.9 eV is higher than that of Zn_2_SnO_4_ [26,27]. Depending on the structure and phase ZTO nanostructures have been used for electronic [22,28,29,30,31] and energy harvesting [14,32,33,34,35,36] devices, catalysis [18,37,38,39,40,41], and sensors [42,43,44].

While vapor phase processes such as chemical vapor deposition (CVD) and thermal evaporation can be used to synthetize ZTO nanostructures with high efficiency [29,45], these expensive techniques are cumbersome and require high temperatures (>700 °C). The demand for low-cost processes compatible with flexible substrates requires solution processes that allow for the synthesis of nanostructures at low cost, using simple and easy methods, ideally upscalable to industrial-scale quantities [46]. Recently, Shaodong et al. reviewed the different methods to fabricate ZTO nanostructures, showing the lack of well-controlled solution based methods to produce ZTO nanowires (NWs) of both ZnSnO_3_ and Zn_2_SnO_4_ phases [12].

The multicomponent nature of ZTO makes the synthesis process quite challenging, given the different ionic sizes and diffusivity of the cations. Furthermore, each ZTO structure has different nucleation and growth times and requires a specific range of synthesis temperature [47]. For these reasons, a comprehensive study on the synthesis of ZTO nanostructures is needed. Studies on the influence of chemico-physical parameters on the hydrothermal synthesis of ZTO have been reported, showing the possibility to control the shape and type of the nanostructures and consequently the electrical, optical, and mechanical properties [13,48,49]. Recently, we reported a thorough description of the influence of different hydrothermal synthesis’ chemical parameters in the growth of ZTO nanostructures [50]. This work was conducted without employing any seed-layer; hence, the obtained structures depend exclusively on the chemico-physical parameters of the synthesis. Moreover, the obtained nanostructures are produced in form of powder, which in conjunction with a variety of transfer methods, allow for a higher degree of freedom for integration on different substrates [51], without contamination from the seed-layer material [44]. In the last decade, the physical parameters of solution-based synthesis have also been studied showing that they have a large influence in the growth of the nanostructures. Zeng et al. reported the influence of temperature and time [49], showing that in order to achieve Zn_2_SnO_4_ nanocrystals, a temperature of 200 °C and at least 20 h of reaction time are needed. Gou et al. studied the evolution of ZnSnO_3_-orth nanoplates with the time and temperature of the synthesis, showing that producing these type of structures required 12 h at 260 °C, for that specific solution process [14]. These structures were applied to nanogenerators, in a composite with PDMS, resulting in a piezoelectric coefficient (d33) of 49 pC/N. This value is more than three times than the typically reported for ZnO nanostructures [52].

In this paper, the effect of the physical parameters on the hydrothermal synthesis of ZTO nanostructures, namely the influence of the volume of solution, temperature, and reaction time of the synthesis, is shown. In line with our previous work about the effect of the chemical conditions, this study is orientated towards the synthesis of ZTO nanowires, more specifically ZnSnO_3_, which properties allow to envisage application on numerous next-generation nanoscale devices such as nanogenerators [35,36,53], sensors [54,55,56], photocatalysis [57], solar cells [58], resistive switching memories [59,60], and transistors [22,29]. Moreover, we have previously shown that the structures synthetized using this hydrothermal method have properties that are similar to those produced by expensive and high-temperature methods [50], thus, being promising materials for a new wave of multifunctional and low-cost devices.

## 2. Materials and Methods

### 2.1. Nanostructures’ Synthesis

ZnSnO_3_ nanowires were synthesized via hydrothermal method in a conventional oven, using the same methodologies and reagents reported previously by our group [50]. The most favorable chemical conditions to produce ZnSnO_3_ nanowires previously obtained in [50] using tin (IV) chloride 5-hydrate (SnCl_4_·5H_2_O) as tin precursor and both zinc chloride (ZnCl_2_) and zinc acetate (Zn(CH_3_COO)_2_, ZnAc) as zinc precursors were maintained in this work. Briefly the ZTO hydrothermal synthesis was performed by dissolving the zinc (0.02 M/0.0657 g of ZnAc or 0.04 M/0.0818 g of ZnCl_2_) and tin (0.02 M/0.1050 g) precursors separately in 7.5 mL of deionized water and then mixed together, 7.5 mL of the surfactant ethylenediamine (EDA) were then added and left stirring for 30 min, after which the mineralizer sodium hydroxide (NaOH, 0.24 M/0.1450 g) was added. The solution was then transferred into a 45 mL Teflon-lined stainless-steel autoclave (Parr Acid Digestion Bombs, no 4744, Moline, IL, USA), and kept in an electric oven (Thermo Scientific, Waltham, MA, USA) varying the temperature and the reaction time, using a heating ramp of 200 °C/h. Aiming to study the influence of the physical parameters in the ZTO nanostructures growth, we varied the mixture volume (7.5 mL, 11 mL, and 15 mL), the synthesis temperature (150 °C, 180 °C, 200 °C, and 220 °C), and reaction time (2 h, 8 h, 12 h, 18 h, 24 h, 36 h, and 48 h). After the synthesis time, the autoclave was cooled to ambient temperature, naturally. The final product (white precipitate) comprising the nanostructures, was alternately washed with deionized water and isopropyl alcohol (at least 5 times) and centrifuged at 4000 rpm. After washed, the nanostructures were dried at 60 °C, in vacuum, for 2 h. As was previously reported [50], the syntheses present a good reproducibility, especially when using ZnCl_2_ due its better solubility in ethylenediamine.

### 2.2. Nanostructures’ Characterization

All the nanostructures’ characterization was performed with the synthesis product in powder form. In order to study the morphology and elemental composition of the nanostructures, scanning electron microscopy (SEM) and energy dispersive X-ray spectroscopy (EDS) inside an AURIGA CrossBeam workstation were performed (Zeiss, Oberkochen, Germany). The structural characterization was carried out by X-Ray diffraction (XRD) using X’Pert PRO MRD diffractometer (PANalytical, Royston, UK) with Cu Kα radiation and the data acquisition range was 10–90° (2θ) with a step size of 0.033°. Fourier-transform infrared (FTIR) spectra were acquired in the range of 4000–525 cm^−1^ with 4 cm^−1^ resolution and 45° incident angle. The data was recorded using a Smart iTR attenuated total reflectance (ATR) sampling accessory (Thermo Scientific, Waltham, MA, USA) equipped with a single bounce diamond crystal on a Thermo Nicolet 6700 Spectrometer (Thermo Scientific, Waltham, MA, USA). Raman spectra were acquired using an inVia Reflex micro-Raman spectrometer (Renishaw, Wotton-under-Edge, UK) equipped with an air-cooled CCD detector and a HeNe laser using a 532 nm laser excitation with a power of 50 mW, with 0.3 cm^−1^ resolution. All measurements were obtained with an intensity of 50 µW at room temperature in a range of 100–1600 nm, using an integration time of 2 scans (10 s each).

## 3. Results and Discussion

### 3.1. Reaction Mixture Volume

A hydrothermal process is a method to produce single and polycrystalline structures, in aqueous solution at high temperature and high pressure. Both reaction mixture volume and temperature are determinant to the pressure inside the autoclave, consequently defining the growth of the nanocrystals. However, the phase transformation mechanism that occurs in polymorphs materials under high pressure is not completely controlled and understood [61].

In order to study the influence of the pressure caused by the solution’s volume in the ZnSnO_3_ nanowires growth in this specific hydrothermal method, volumes of 7.5 mL, 11 mL, and 15 mL were tested, representing 17%, 24%, and 33% of the autoclave volume, respectively. The temperature was fixed at 200 °C and the reaction time was 24 h.

**Figure 1:**
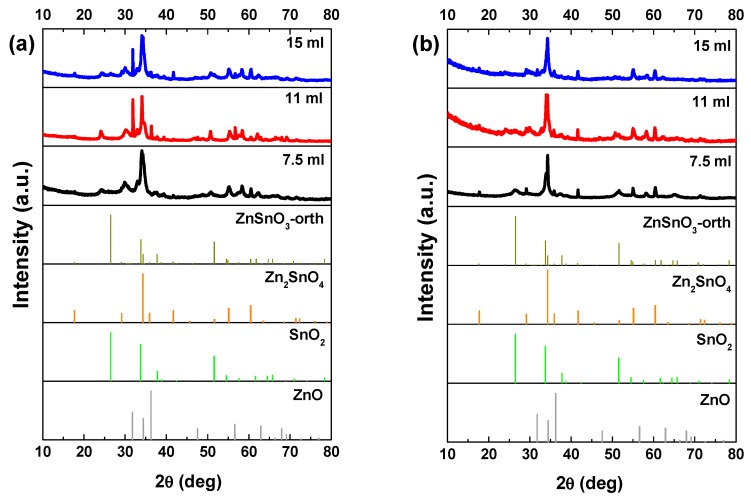
XRD patterns when using (**a**) ZnCl_2_ precursor (with 2:1 Zn:Sn ratio) and (**b**) ZnAc precursor (with 1:1 Zn:Sn ratio), for different solution mixture volumes (7.5 mL, 11 mL, and 15 mL). Identification following ICDD card 00-028-1486 (ZnSnO_3_-orth, deleted), 00-024-1470 (Zn_2_SnO_4_), 01-077-0452 (SnO_2_), and 00-06-1451 (ZnO).

**Figure 2:**
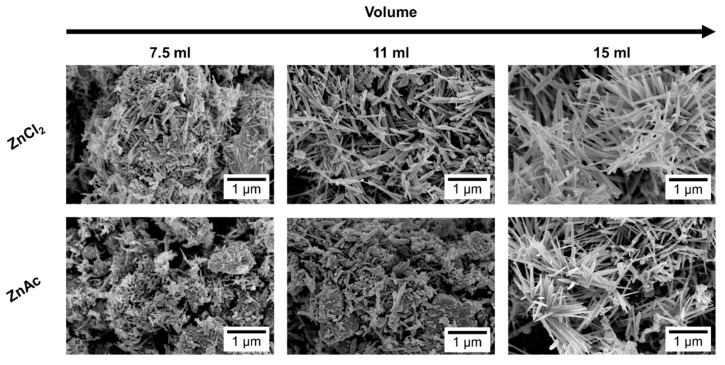
SEM images of nanostructures obtained for different reaction mixture volumes, 7.5 mL, 11 mL, and 15 mL, using ZnCl_2_ and ZnAc as zinc precursor.

In general, a mixture of ZnO nanowires, SnO_2_ nanoparticles, and Zn_2_SnO_4_ nanostructures can be found in these syntheses, still with the increasing of the mixture volume mainly ZnSnO_3_ nanowires are obtained. Figure 1a shows small differences in the XRD pattern for the different volumes when using the ZnCl_2_ precursor. The most significant difference is the peak at 32° appearing for the volumes of 11 and 15 mL, attributed to ZnO phase. Raman analysis (Figure S1a) shows an intense peak at 437 cm^−1^, characteristic of ZnO [62], being this peak more intense for 11 mL, in agreement with the XRD data. The SEM images in Figure 2 clearly show that less volume gives rise to smaller nanowires. As we previously identified in Reference [50], when a volume of 15 mL was used, these nanowires have the ZnSnO_3_ orthorhombic perovskite phase, represented by the card 00-028-1486. Even if this card has been used before in several reports [63,64,65,66], it was deleted from ICDD database due to matching peaks with the mixture of SnO_2_ and Zn_2_SnO_4_. Although in 00-028-1486 card the peak with most intensity is at 26°, this peak corresponds to the 012 plane, which is not the preferential orientation of the nanowires when measured in XRD. In order to confirm the ZnSnO_3_ identification, we performed peak indexing in different samples, which always showed matching to an orthorhombic phase characteristic of ZnSnO_3_ and not of Zn_2_SnO_4_ and SnO_2_ mixture. EDS analysis confirms the 1:1 Zn:Sn ratio of the nanowires (Figure S2), reinforcing the hypothesis of the ZnSnO_3_ phase. Nevertheless, Raman analysis suggests that both ZnSnO_3_ and Zn_2_SnO_4_ should be present in the sample, given the characteristic peaks identified at 538 cm^−1^ and 676 cm^−1^ [49].

When ZnAc is used as the zinc source the presence of ZnO is partially or even completely suppressed. Similar to the results obtained using ZnCl_2_, longer ZnSnO_3_ nanowires are obtained for higher volumes of solution mixture. While for the lower volume (7.5 mL) Zn_2_SnO_4_ is the predominant phase, for 11 mL, ZnSnO_3_ nanowires are predominant, although some Zn_2_SnO_4_ nanostructures are also observed (Figure S3) and ultimately for 15 mL, ZnSnO_3_ nanowires are the predominant structures. SnO_2_ can also be identified by XRD (Figure 1b), even if it is not clear by Raman (Figure S1b) for all the conditions. The presence of residual ZnO/SnO_2_ for synthesis using the ZnCl_2_/ZnAc precursors, is attributed to the Zn precursor’s higher/lower solubility in ethylenediamine when compared to the SnCl_4_.5H_2_O, leading to an earlier availability of Zn/Sn species in the synthesis [50].

Nakayama et al. reported a theoretical study where the relation between the enthalpy and pressure for the system Zn–Sn–O was investigated [23]. They showed that the global energy minimum corresponds to the mixed phases Zn_2_SnO_4_ and SnO_2_. Our experimental results are in agreement with this study, as for lower volume and consequently lower pressure, the formation of Zn_2_SnO_4_ and SnO_2_ nanoparticles is favored, although mixed with ZnSnO_3_ nanowires. Moreover, in 2010 Gou et al. also reported a theoretical study about the ZnSnO_3_ phase transition under pressure [61]. According to the authors the ZnSnO_3_ synthesis at low-temperature (<700 °C) is unfavorable, leading to a mixture of phases, mainly due to its positive formation enthalpies. This decomposition has also been reported to occur at temperature above 500 °C [13,67]. Taking into account the temperature used in this study (200 °C), our results are in fair agreement with this, since with decreasing volume, and consequently pressure, the trend is to achieve higher mixture of phases while the more homogeneous ZnSnO_3_ nanowires are obtained when using the higher solution volume, 15 mL. The high pressure inside the autoclave, characteristic of a hydrothermal method, supplies high energy levels to the reaction, leading to an acceleration of the nucleation processes at even lower temperatures. For the reasons explained, 15 mL was the volume used for both precursors for the next studies presented here.

### 3.2. Synthesis Temperature

To understand the growth mechanisms of ZTO nanostructures, one needs to have in mind the chemical reaction processes. Based on different reports, the ZnSnO_3_ nanostructures formation follows the equations below [14,63,68]:

Zn^2+^ + Sn^4+^ + 6OH^−^ → ZnSn(OH)_6_, (1)

ZnSn(OH)_6_ → ZnSnO_3_ + 3H_2_O. (2)

Regarding Zn_2_SnO_4_, its formation can be represented by the following equations together with Equation (1):

Zn^2+^ + 4OH^−^ → Zn(OH)_4_^2−^, (3)

ZnSn(OH)_6_ + Zn(OH)_4_^2−^ → Zn_2_SnO_4_ + 4H_2_O + 2OH^−^ (4)

ZnSnO_3_ can have the fcc or the orthorhombic structures, and these two different structures present different heat of formation and total energies. Gou et al. presented these values for the different possible structures of ZnSnO_3_ [61]. On the other hand, it is already well-known that the ZnSnO_3_ phase is metastable [12,13] and according, for example, to the study reported by Bora et al. this phase suffers a decomposition into the thermodynamically stable phases Zn_2_SnO_4_ and SnO_2_ at temperatures higher than 500 °C [67]. This decomposition can be represented by the following equation:

2ZnSnO_3_ → Zn_2_SnO_4_ + SnO_2_. (5)

However, it is clear that above atmospheric pressure, as in the case of the hydrothermal method, and depending of the reaction mixture, the temperature at which the decomposition occurs can change.

In order to investigate the effect of temperature in the specific conditions of our syntheses, four different temperatures were used: 150 °C, 180 °C, 200 °C, and 220 °C, while maintaining all the other conditions and keeping the volume of the solution mixture at 15 mL.

**Figure 3:**
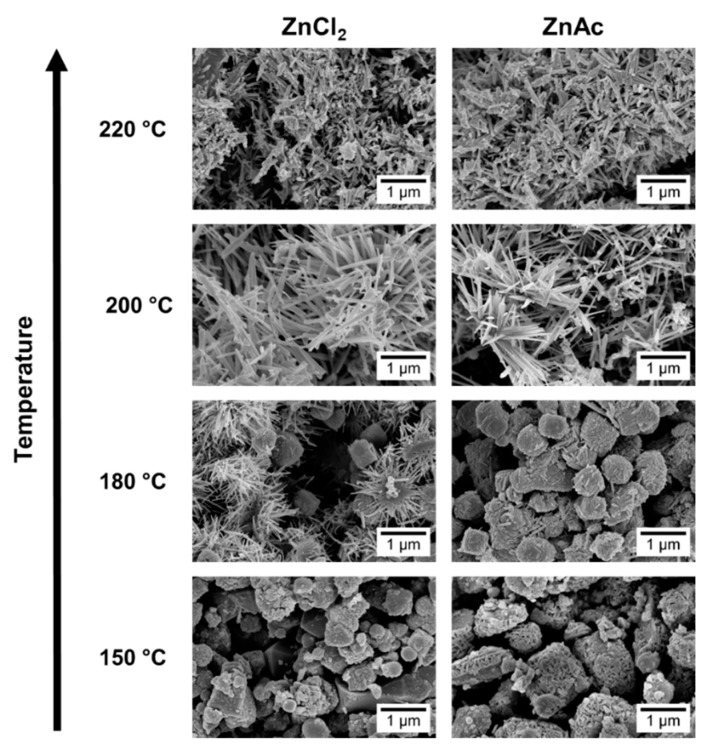
SEM images of the nanostructures obtained using the previous best conditions (15 mL) for each zinc precursor (ZnCl_2_ and ZnAc) at different temperatures: 150 °C, 180 °C, 200 °C, and 220 °C.

**Figure 4:**
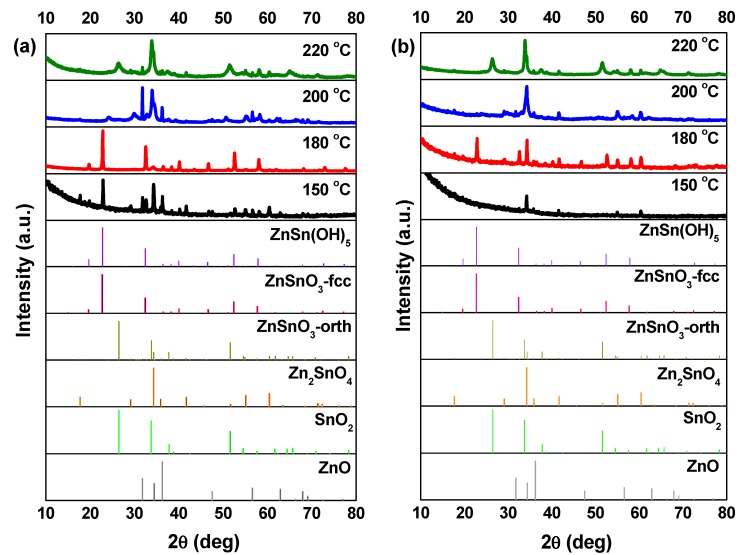
XRD patterns when using (**a**) ZnCl_2_ precursor (with 2:1 Zn:Sn ratio) and (**b**) ZnAc precursor (with 1:1 Zn:Sn ratio), at different temperatures: 150 °C, 180 °C, 200 °C, and 220 °C. Identification following ICDD card 00-028-1486 (ZnSnO_3_-orth—deleted), 00-011-0274 (ZnSnO_3_-fcc), 00-024-1470 (Zn_2_SnO_4_), 01-077-0452 (SnO_2_), and 00-06-1451 (ZnO).

SEM images, in Figure 3, show that at 150 °C, mainly nanoparticles are obtained. In the XRD pattern (Figure 4) a mixture of phases (ZnSn(OH)_6_ and Zn_2_SnO_4_ and/or ZnSnO_3_) can be observed using ZnCl_2_, while when using ZnAc, only Zn_2_SnO_4_ is identified. Raman analysis (Figure S4) is in agreement with these results, showing the peak at 603 cm^−1^, characteristic of ZnSn(OH)_6_ (only for ZnCl_2_) and the characteristics peaks of ZTO for both Zn sources (538 cm^−1^ and 676 cm^−1^). It is important to refer that the 603 cm^−1^ vibrational band is not only a characteristic peak of ZnSn(OH)_6_, but also of fcc-ZnSnO_3_ [67] that could also be present in these samples. However, FTIR analysis supports the ZnSn(OH)_6_ hypothesis by showing OH^−^ groups for wavenumbers above 3000 cm^−1^ (Figure S5a), corresponding to ZnSn(OH)_6_. In EDS analysis, shown in Figure S6, it can be seen that the nanoparticles obtained using ZnAc have the Zn:Sn ratio of 2:1 corresponding to the Zn_2_SnO_4_ phase.

Increasing the temperature to 180 °C, some nanowires can be viewed in SEM when using the ZnCl_2_ precursor (Figure 3), which can be ZnSnO_3_ according to the XRD (Figure 4a). Still, at this temperature, OH^−^ groups are very evident from the FTIR analysis (Figure S5a) for both zinc precursors, with XRD showing predominantly the ZnSn(OH)_6_ phase, an intermediate stage of the ZnSnO_3_ formation. Zeng et al. also showed that for temperatures lower than 200 °C ZnSn(OH)_6_ is the predominant phase [49]. Nevertheless, XRD also shows that other phases are present (Figure 4). In fact, EDS (Figure S7) and Raman (Figure S4) confirm this by showing a reasonable amount of Zn_2_SnO_4_ octahedrons, especially when using ZnAc as precursor. It means that, as already discussed, at these lower temperatures, and consequently lower pressures, the formation of the thermodynamically more stable Zn_2_SnO_4_ nanoparticles is promoted. The lower solubility of ZnAc in ethylenediamine, when compared to ZnCl_2_, can explain why a higher mixture of phases is seen for this case.

Moreover, in Figure S5a,b, FTIR spectra of both precursors for 150 °C and 180 °C presents several peaks in the range of 1250 to 500 cm^−1^. The origin of these peaks can be associated to residual reagents in the final product (see Figure S8 in supporting information) [69], which means that when using temperatures lower than 200 °C, the reagents are not completely consumed. An exception is seen for synthesis at 150 °C using ZnAc, where these peaks are not present and Zn_2_SnO_4_ nanoparticles were obtained, as these are the more stable ZTO nanostructures.

The samples at 200 °C were already described in the previous section and result mainly in ZnSnO_3_ nanowires. For this temperature, as seen in Figure S5, FTIR analysis confirms that no OH^−^ groups are present, meaning that the intermediate phase ZnSn(OH)_6_ is no longer obtained at this temperature. It should be added that FTIR also shows that all the reagents were completely consumed.

Increasing the temperature to 220 °C leads to the formation of SnO_2_ and Zn_2_SnO_4_ phases, even if the ZnSnO_3_ nanowires are still predominant. While it is not evident in the XRD pattern, through Raman analysis (Figure S4) their presence is clear since it is possible to observe the 631 cm^−1^ and 538 cm^−1^ peaks, characteristic of Sn–O and M–O tetrahedrons bonds, respectively. This result is attributed to the decomposition of ZnSnO_3_ into SnO_2_ and Zn_2_SnO_4_, due to its metastability. This decomposition has been reported to occur at temperatures above 500 °C [13,61,67]. In the present study, this decomposition occurs at lower temperature due to the high energy levels inherent to the hydrothermal process (high temperature and pressure).

In conclusion, it is clear that for temperatures lower than 200 °C, homogeneous nanowires are not produced as, in accordance to what was observed in the previous section, for lower available energy, the formation of ZnSnO_3_ is not feasible, and instead the more stable phase Zn_2_SnO_4_ is favored (along with the intermediate phase ZnSn(OH)_6_). Meanwhile, for temperatures around 200 °C, almost only ZnSnO_3_ nanowires are produced. For higher temperatures (220 °C), no improvements are observed in terms of producing only nanowires, as decomposition of those into the more stable phases starts to occur. This study shows that the temperature to achieve the desired structures must be carefully considered, as it should be high enough to allow the formation of the metastable phase, but not too high to promote the decomposition process.

### 3.3. Reaction Time

Throughout the reaction time of the synthesis, the ZTO nanostructures undergo a complex evolution. Thus, a careful investigation on the evolution of ZTO morphology and crystallinity was performed for different reaction times (2 h, 8 h, 12 h, 18 h, 24 h, 36 h, and 48 h), for both zinc precursors (ZnCl_2_ and ZnAc) and using the most favorable conditions as defined in the previous sections (15 mL of volume at 200 °C).

Similar to what was observed with the study of the effect of the temperature, ZnSn(OH)_6_ appears as an intermediate phase before the formation of the ZnSnO_3_ or the Zn_2_SnO_4_ phases (represented by Equations (1)–(4)).

**Figure 5:**
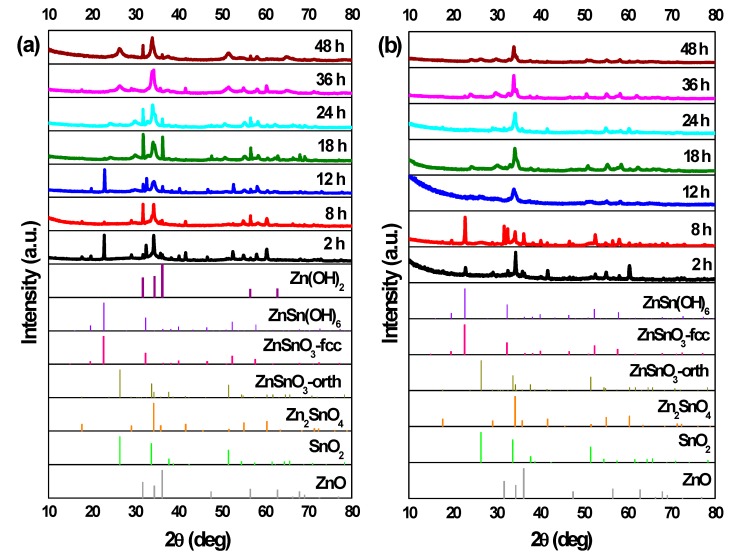
XRD patterns when using (**a**) ZnCl_2_ precursor (with 2:1 Zn:Sn ratio) and (**b**) ZnAc precursor (with 1:1 Zn:Sn ratio), for different reaction time: 2 h, 8 h, 12 h, 18 h, 24 h, 36 h, and 48 h. Syntheses at 200 °C, using 15 mL of reaction mixture volume. Identification following ICDD cards: 00-028-1486 (ZnSnO_3_-orth—deleted), 00-011-0274 (ZnSnO_3_-fcc), 00-024-1470 (Zn_2_SnO_4_), 01-077-0452 (SnO_2_), 00-06-1451 (ZnO), and 00-048-1066 (Zn(OH)_2_).

In Figure 5a,b, the XRD patterns for the structures from synthesis with different reaction times are shown. For 2 h of synthesis, ZnSn(OH)_6_ can be identified, for both zinc precursors. As already mentioned, this phase can be mistaken with fcc-ZnSnO_3_ in XRD pattern and also in Raman analysis (Figure S9) where an intense peak at 603 cm^−1^ is evident. Nonetheless, FTIR analysis in Figure S10 clarifies this by showing the presence of OH^−^ groups, confirming the presence of ZnSn(OH)_6_. Although ZnSn(OH)_6_ phase is predominant for both Zn sources, when using ZnAc, it is also possible to identify Zn_2_SnO_4_ by XRD, and when using ZnCl_2,_ Raman analysis indicates ZnSnO_3_ and/or Zn_2_SnO_4_ may be present.

When using ZnCl_2_, the ZnSn(OH)_6_ phase appears for synthesis of 2 h, while for synthesis of 8 h or longer, neither OH^−^ groups (from the intermediate phase ZnSn(OH)_6_) nor leftover precursors are observed in FTIR spectra (Figure S10), unlike when using ZnAc, where these are present up to 8 h of synthesis. For 8 h of synthesis, while the nanoparticles are still predominant, some orthorhombic ZnSnO_3_ nanowires start to be present and, from this reaction time onwards, these nanowires are the predominant structure achieved, with both precursors.

Regarding the use of the ZnCl_2_, a particular case is observed for the 12 h long synthesis. In this condition, ZnSnO_3_-fcc also seems to be obtained, mixed with ZnSnO_3-_orth. The possibility to obtain this type of structures for lower energies was already demonstrated as it is an intermediate phase between ZnSn(OH)_6_ and ZnSnO_3_-orth [61]. Also, it is relevant to consider the possibility of formation of Zn(OH)_2_ due to the presence of Zn(OH)_4_^2+^, which is an intermediate phase of formation of both Zn_2_SnO_4_ and ZnO. In XRD, the phase Zn(OH)_2_ (that is transformed in Zn(OH)_4_^2+^) can be mistaken with hexagonal phase ZnO. Also, in EDS mapping (shown in Figure S11), it is possible to observe some nanoparticles with a large Zn signal, that can be attributed either to Zn(OH)_2_ or to Zn_2_SnO_4_. As such, the possibility of having Zn(OH)_2_ cannot be discarded. This emphasizes the difficulty of obtaining only one phase and one type of ZTO nanostructure using a solution-based method and the demand for a well-controlled synthesis process.

**Figure 6:**
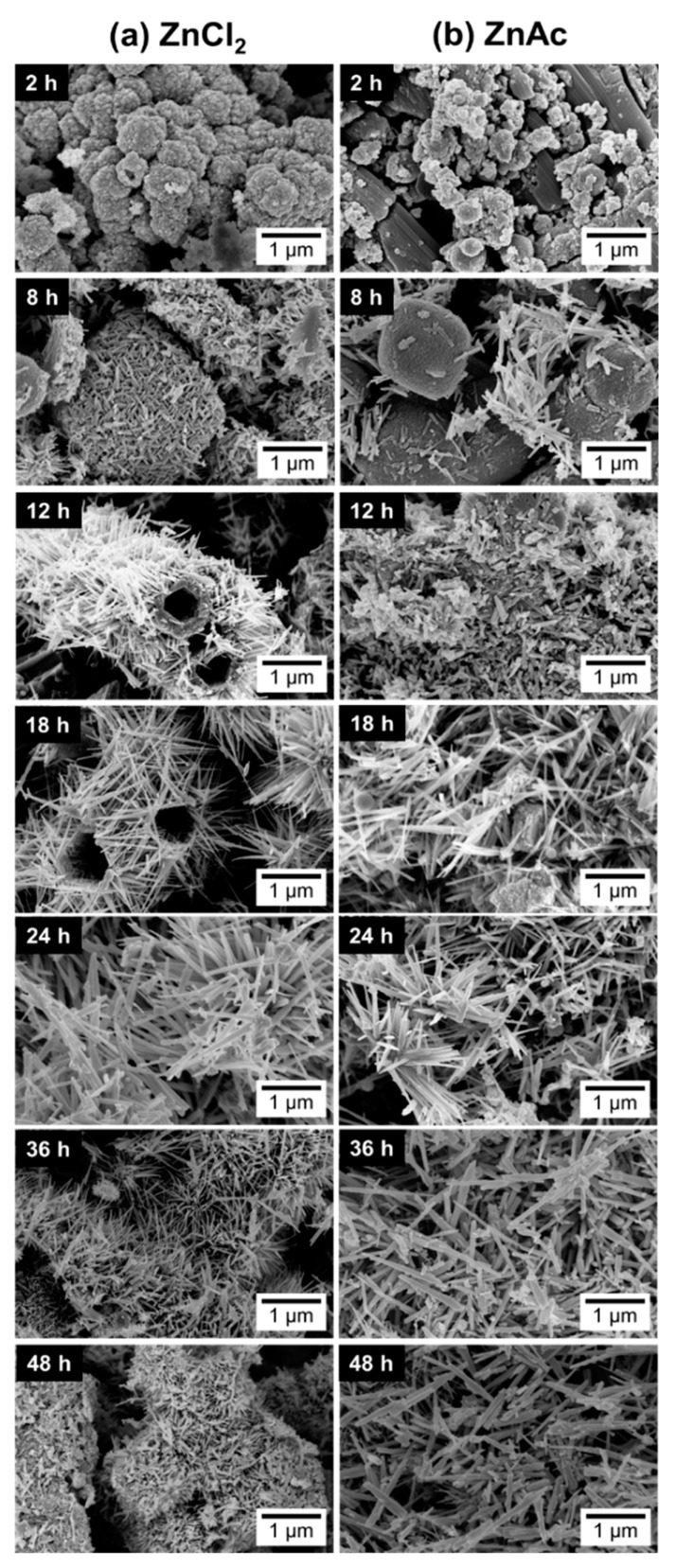
SEM images of the nanostructures obtained by synthesis using ZnCl_2_ (**a**) and ZnAc (**b**) with different reaction time, showing the temporal evolution of the structures produced. Syntheses at 200 °C, using 15 mL of reaction mixture volume.

In the case of using the ZnAc precursor, for synthesis of 12 h or longer, the XRD pattern (Figure 5a) seems to be similar (evidencing ZnSnO_3_ nanowires) with a higher crystallinity for the longer synthesis when compared to 12 h. SEM images (Figure 6) show that longer nanowires are obtained for 36 h or 48 h long synthesis. Table 1 shows the lengths of the nanowires for both precursors, emphasizing this observation. On the other hand, when using ZnCl_2_, while nanowires’ lengths increase up to around 18 h or 24 h, these start to significantly decrease for longer synthesis (Figure 6, Table 1). It can be observed through XRD data (Figure 5) that when synthesis is 36 h or longer, ZnSnO_3_ phase transformation into Zn_2_SnO_4_ and SnO_2_ starts to occur, in a similar trend to that seen in the previous section for the increase of temperature beyond 200 °C, causing the diminishing of the nanowires’ length.

It can be induced that, due to the ZnCl_2′_s higher solubility in ethylenediamine, the evolution of the phases happens faster when this precursor is used. While for ZnCl_2_, a phase transformation of ZnSnO_3_ to Zn_2_SnO_4_ and SnO_2_ starts to occur at 36 h, for ZnAc, a pronounced length increase is still observed up to 36 h, supporting the assumption that a slower reaction is taking place when using ZnAc precursor. While this results in an increased synthesis time to achieve longer nanowires, these are significantly longer (more than 2x) than when using ZnCl_2_. Nevertheless, for 48 h, there is a small deterioration in terms of nanowire length and homogeneity of the nanostructures’ phases, thus 36 h is the optimal reaction time for the conditions studied.

In summary, in Figure 6, one can clearly visualize the large dependency of the nanostructures’ growth with the increase of reaction time. For both precursors, it is clear that at least 12 h are necessary to start producing predominantly ZnSnO_3_ nanowires, being that only after 24 h this production starts being homogeneous. It is also possible to observe that, similarly to what was concluded with previous studies on the influence of the chemical parameters [50], the ZTO nanostructures obtained using ZnAc are generally smaller than those obtained using ZnCl_2_, for synthesis up to 24 h. When using ZnAc, the ZTO growth is slower so the higher dimensions are obtained for longer reaction times (36 h and 48 h), for which the nanowires reach higher dimensions than that those obtained with ZnCl_2_. This difference in the optimal reaction time can be attributed to the lower solubility of ZnAc when compared with ZnCl_2_ in the solvent ethylenediamine. This also leads to a constant presence of SnO_2_ for the syntheses with ZnAc, while when using ZnCl_2_, ZnO is constantly observed. This study shows that if longer nanowires are desired, ZnAc is the most suitable precursor, while if faster processing is desired, the most suitable precursor is ZnCl_2_.

### 3.4. Growth Mechanism of ZnSnO_3_ Nanowires

Throughout the reaction time one of the main challenges in nanomaterials synthesis is to understand how the nanostructures grow, i.e., what are the mechanisms behind the formation of each phase and of each type of nanostructures. Obtaining only one phase and one shape is not an easy task, particularly for the Zn:Sn:O system due to its ternary composition, as evident throughout this manuscript. In the specific case of ZnSnO_3_ nanowires formation, the metastability of this phase adds even more complexity into understanding it properly. Gou et al. showed the influence of pressure and temperature in the enthalpy energy of different ZTO phases, including ZnSnO_3_ cubic perovskite, concluding that the formation of this phase is not favorable under ambient conditions and can only be obtained under high pressure and temperature [61]. However, under those conditions, this phase tends to decompose into Zn_2_SnO_4_ and SnO_2_ [70]. Therefore, a very specific tailoring of the synthesis parameters is necessary when aiming to achieve proper structures of this metastable phase.

**Figure 7:**
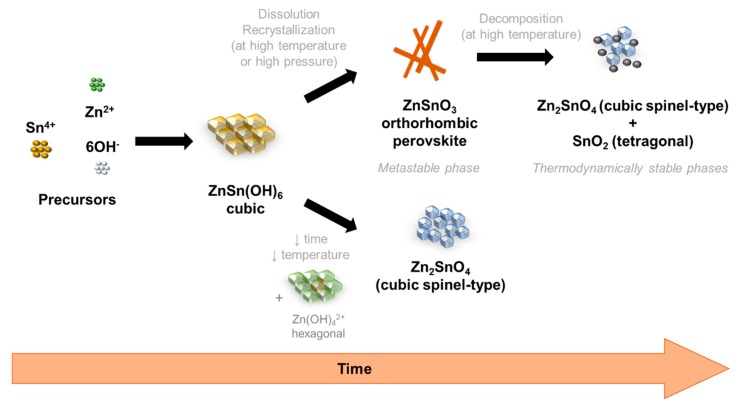
Schematic representation of the phase transformations on the hydrothermal synthesis of zinc-tin oxide (ZTO) nanostructures, depending on the energy available in the reaction and the time of synthesis.

Owing to the careful optimization of the chemico-physical parameters performed here, it is possible to observe an evolution of the nanostructures, and its respective phase, with the increase of reaction time and increase of total energy available in the reaction. Although the aim was to achieve ZnSnO_3_ nanowires, Zn_2_SnO_4_ nanostructures were also obtained, mainly for very short synthesis or for synthesis with lower temperatures or volumes, showing that this phase is achieved with lower available energy. These trends are represented in the Figure 7.

## 4. Conclusions

Due to its complexity, the synthesis of multicomponent oxide nanostructures requires an appropriate understanding of the influence of each synthesis’ parameters and the growth mechanism itself. In this work, a detailed study on the influence of physical parameters on the hydrothermal synthesis of ZnSnO_3_ nanowires is reported. Specifically, the effect of reaction mixture volume, synthesis temperature and reaction time were studied. The available energy in the reaction revealed to be one of the determinant factors on the final products. As already reported in the literature, the metastable ZnSnO_3_ requires high pressures/energy conditions to be obtained. It was found that independently of the Zn source, ZTO nanostructures had the same time-dependence growth mechanisms, although with different time spans. 

While previous reports have shown the degradation of the metastable ZnSnO_3_ structures at temperatures above 500 °C, the present work demonstrates that owing to the high energy of the hydrothermal process, this decomposition can occur at considerably lower temperatures (24 h at 220 °C) and/or longer reaction times (36 h at 200 °C). This highlights the advantages of hydrothermal processes to obtain metastable multicomponent nanostructures such as ZnSnO_3_ at low temperatures but also the importance of properly controlling and understanding all the synthesis parameters to achieve the desired structures. With ZnSnO_3_ finding application as a piezoelectric material, catalyst, active material in gas sensors, resistive switching memories, batteries, and others, we expected this work to have a significant impact in the future of nanotechnology as it describes the synthesis of ZnSnO_3_ nanowires without either direct growth in substrates or production by vapor phase methods (high temperatures). This new approach brings higher flexibility to the different applications, allowing a higher degree of freedom for integration on different substrates while avoiding contamination from the seed-layer material.

## Figures and Tables

**Table 1:** Evolution of the average of nanowires’ length with the reaction time.

*Reaction Time (h)*	*Nanowires’ Length (nm)*	
	ZnCl_2_	ZnAc
24	605 ± 75	540 ± 175
36	420 ± 115	1090 ± 250
48	220 ± 35	950 ± 190

## Supplementary Materials

The following are available online at https://www.mdpi.com/2079-4991/9/7/1002/s1, Figures S1, S4, and S9: Raman spectroscopy of the samples related to the studies of volume, temperature, and reaction time, respectively. Figures S2, S3, S6, S7, and S11: SEM images and EDS element analysis, which is used to assist in phases identification of each structure. Figures S5, S8, and S10: FTIR reaction time, synthesis temperature, and all reagents used in the syntheses, respectively.
