# Supplementary material for: Growth Mechanism of Seed-Layer Free ZnSnO3 Nanowires: Effect of Physical Parameters
Source: Nanomaterials (Basel). 2019 Jul 11;9(7):1002. doi: 10.3390/nano9071002 (PMC6669656; doi:10.3390/nano9071002)
Supplement: Supplementary file 1 [file nanomaterials-09-01002-s001.pdf]

Supporting Information

Growth Mechanism of Seed-Layer Free $ZnSnO_3$ Nanowires: Effect of Physical Parameters

Ana Rovisco, Rita Branquinho, Jorge Martins, Elvira Fortunato, Rodrigo Martins and Pedro Barquinha**

CENIMAT/i3N Departamento de Ciência dos Materiais, Faculdade de Ciências e Tecnologia (FCT), Universidade NOVA de Lisboa (UNL), and CEMOP/UNINOVA, 2829-516 Caparica, Portugal. *E-mail: a.rovisco@campus.fct.unl.pt, pmcb@fct.unl.pt

The supporting information contains relevant data related to the characterization of the synthetized ZTO nanostructures. In the Figures S1, S4 and S9 Raman spectroscopy of the samples related to the studies of volume, temperature and reaction time, respectively, is shown. Figures S2, S3, S6, S7 and S11 show SEM images and EDS element analysis, which is used to assist in phases identification of each structure. Figures S5, S8 and S10 show FTIR spectra of reaction time, synthesis temperature and all reagents used in the syntheses, respectively.

1. Reaction mixture volume

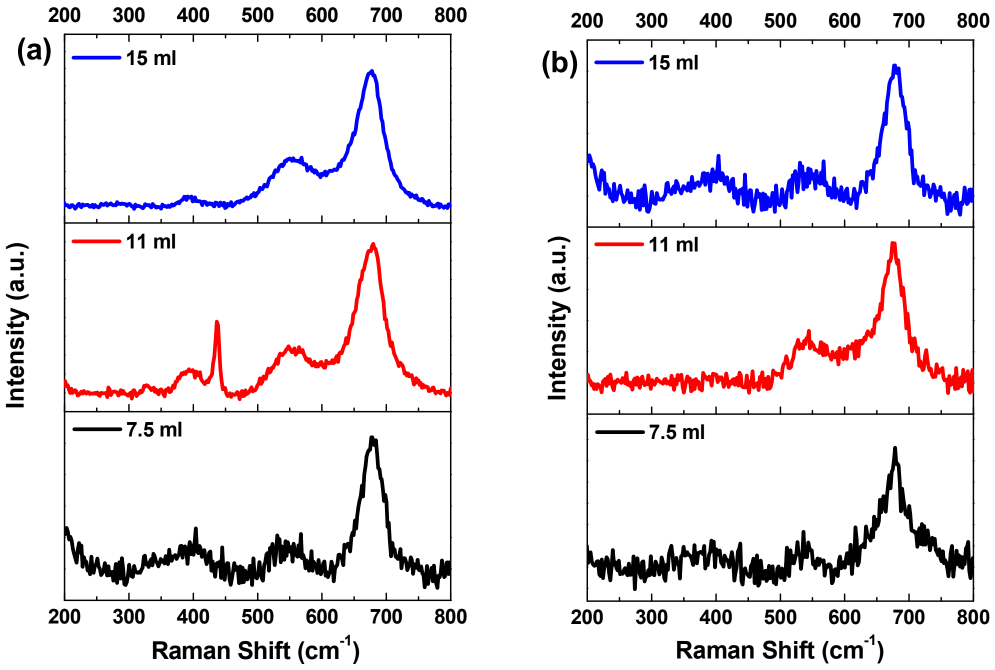

Figure S1. Raman shift of different reaction mixture volumes, 7.5 ml, 11 ml and 15 ml, using (a) $ZnCl_2$ (Zn:Sn molar ratio of 2:1) and (b) ZnAc (Zn:Sn molar ratio of 1:1) as zinc precursor, at 200 °C, for 24 h. Where: vibrational band at 631 cm^{-1} is associated with the expansion and contraction of the Sn–O bond peak, peaks at 538 and 676 cm^{-1} correspond to internal vibrations of the oxygen tetrahedron in Zn_2SnO_4 and to characteristic Raman M–O bonds stretching vibration mode in the MO_6 octahedron of $ZnSnO_3$ and/or Zn_2SnO_4, respectively; and peaks at 437 cm^{-1} and 574 cm^{-1} are attributed to vibrational modes of ZnO [1–3].

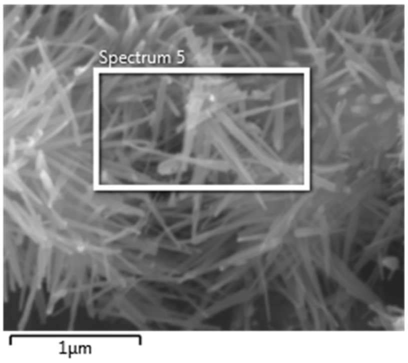

Element	Atomic concentration (%)
**Zn**	15
**Sn**	17
**O**	68

Figure S2. SEM image and EDS element quantification of $ZnSnO_3$ nanowires produced by synthesis using $ZnCl_2$, a Zn:Sn molar ratio of 2:1, a volume 15 ml at 200 °C for 24 h. This analysis shows Zn:Sn ratio of 1:1, supporting identification of the $ZnSnO_3$ phase. The higher than expected atomic concentration of oxygen can be atributted to the carbon tape.

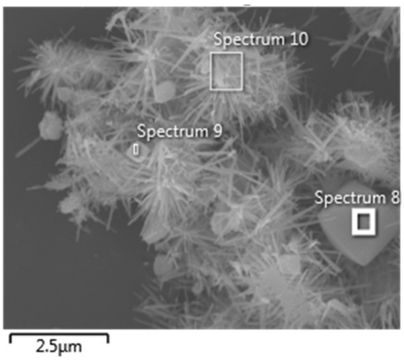

Element	Atomic concentration (%)
**Zn**	29
**Sn**	14
**O**	57

Figure S3. SEM image and EDS element quantification of isolated Zn_2SnO_4 nanostructures produced by synthesis using ZnAc, a Zn:Sn molar ratio of 1:1 using a volume of 11 ml at 200 °C for 24 h.

2. Synthesis temperature

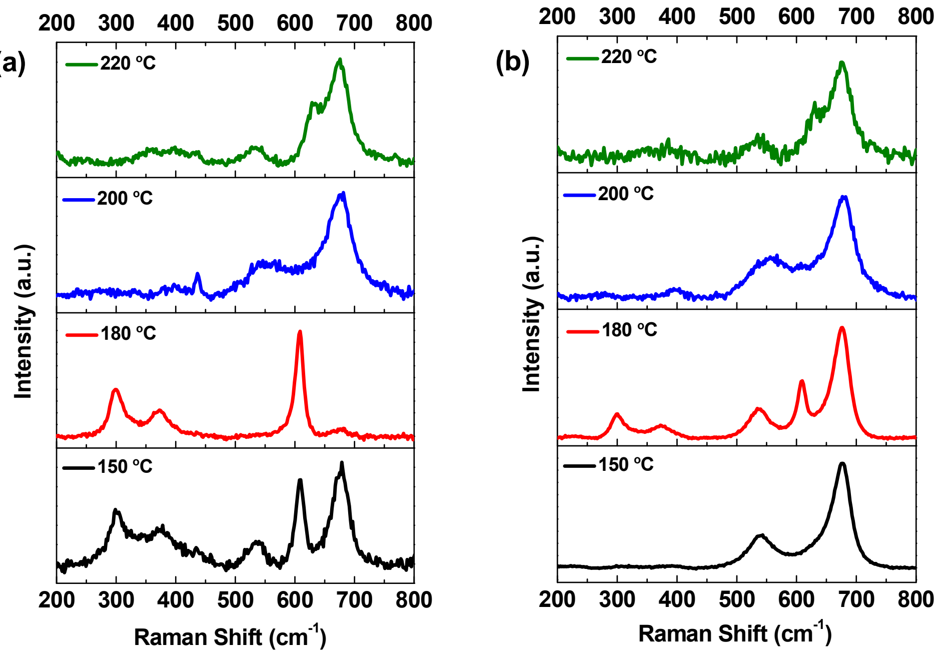

Figure S4. Raman shift for syntheses using (a) $ZnCl_2$ (Zn:Sn molar ratio of 2:1) and (b) ZnAc (Zn:Sn molar ratio of 1:1) as zinc precursor, with a volume of 15 ml, for 24 h at different temperatures: 150 °C, 180 °C, 200 °C and 220 °C. Where: vibrational band at 631 cm^{-1} is associated with the expansion and contraction of the Sn−O bond peak, peaks at 538 cm^{-1} and 676 cm^{-1} are corresponding to internal vibrations of the oxygen tetrahedron in Zn_2SnO_4 and to characteristic Raman M−O bonds stretching vibration mode in the MO_6 octahedron of $ZnSnO_3$ and/or Zn_2SnO_4, respectively; and the peak at 437 cm^{-1} is attributed to vibrational mode of ZnO. The peaks at 299, 372, and 603 cm^{-1} correspond to $ZnSn(OH)_6$, from the breathing vibrations of long M–OH bonds and M–OH–M (bridging OH group) bending modes [1–4].

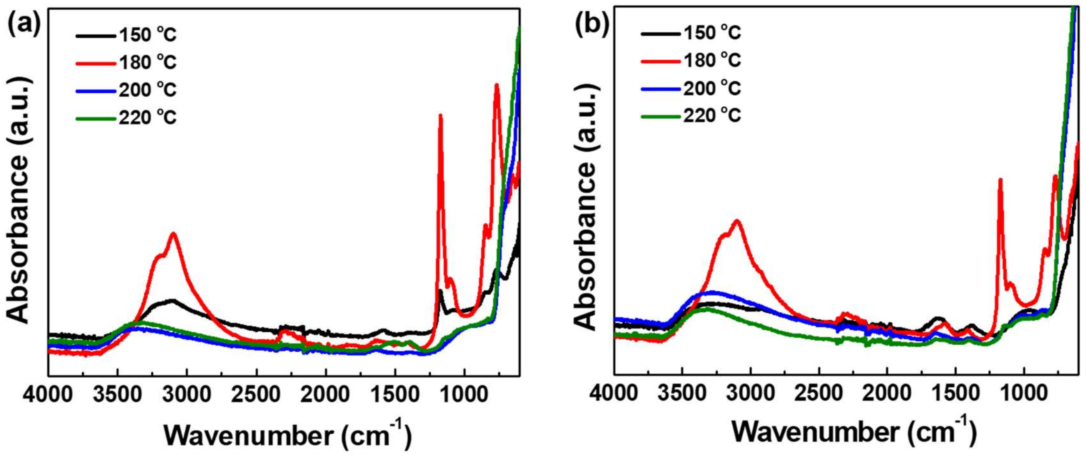

Figure S5. FTIR spectra of the obtained nanostructures using (a) $ZnCl_2$ and (b) ZnAc, at different temperatures (150 °C, 180 °C, 200 °C and 220 °C).

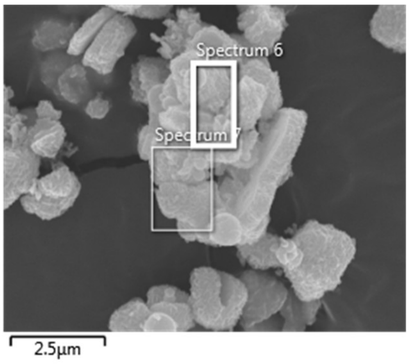

Element	Atomic concentration (%)
**Zn**	21
**Sn**	13
**O**	66

Figure S6. SEM image and EDS element quantification of isolated Zn_2SnO_4 nanostructures produced by synthesis using ZnAc, Zn:Sn molar ratio of 1:1 and a volume of 15 ml at 150 °C for 24 h.

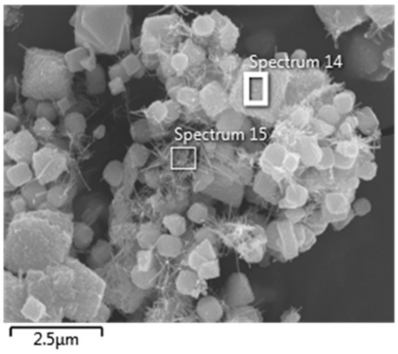

Element	Atomic concentration (%)
**Zn**	26
**Sn**	11
**O**	63

Figure S7. SEM image and EDS element quantification of isolated Zn_2SnO_4 octahedrons produced by synthesis using ZnAc and Zn:Sn molar ratio of 1:1 and a volume of 15 ml at 180 °C for 24 h.

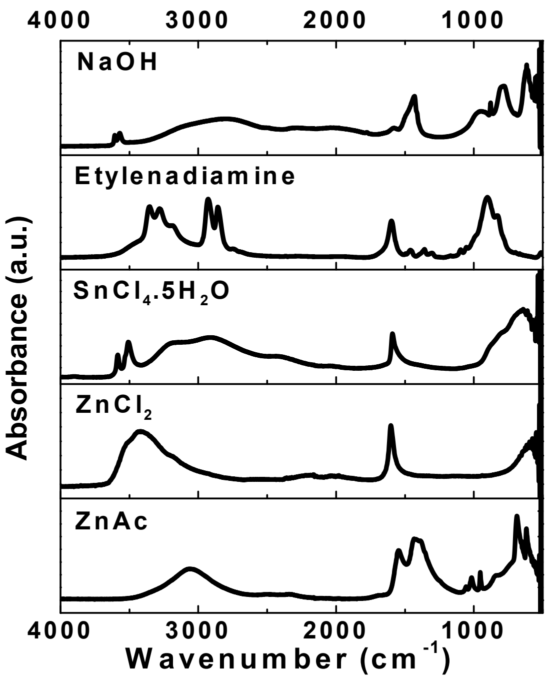

Figure S8. FTIR spectra of all reagents used in the syntheses: the zinc and tin precursors ($ZnCl_2$, $Zn(CH_3COO)_2$ and $SnCl_4{\cdot}5H_2O$), the mineralizer (NaOH) and the surfactant (ethylenediamine).

3. Reaction time

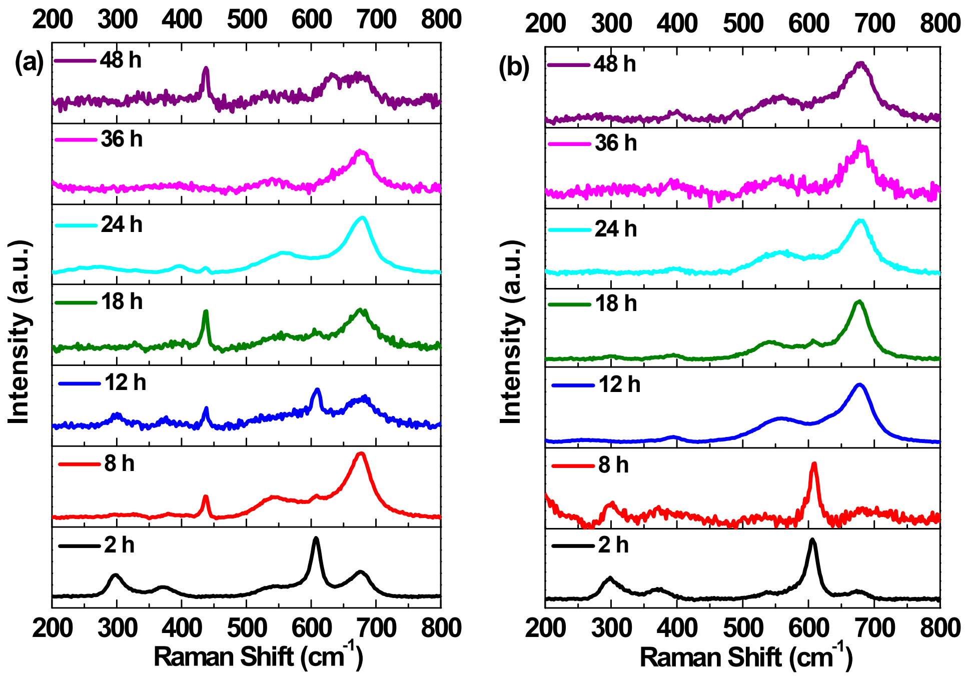

Figure S9. Raman shift for synthesis with different reaction times using as zinc precursor (a) $ZnCl_2$ (Zn:Sn molar ratio of 2:1) and (b) ZnAc (Zn:Sn molar ratio of 1:1), with a volume of 15 ml, at 200 °C. Where: vibrational band at 631 cm^{-1} is associated with the expansion and contraction of the Sn−O bond, peaks at 538 cm^{-1} and 676 cm^{-1} correspond to internal vibrations of the oxygen tetrahedron in Zn_2SnO_4 and to characteristic Raman M−O bonds stretching vibration mode in the MO_6 octahedron of $ZnSnO_3$ and/or Zn_2SnO_4, respectively; and the peak

at 437 cm^{-1} is attributed to vibrational mode of ZnO. The peaks at 299, 372, and 603 cm^{-1} correspond to $ZnSn(OH)_6$, from breathing vibrations of the long M–OH bonds and M–OH–M (bridging OH group) bending modes. [1–4]

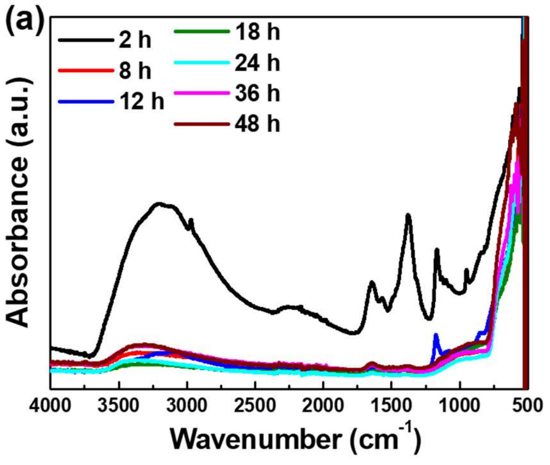

Figure S10. FTIR spectrum of samples with different reaction times for (a) $ZnCl_2$ and (b) ZnAc.

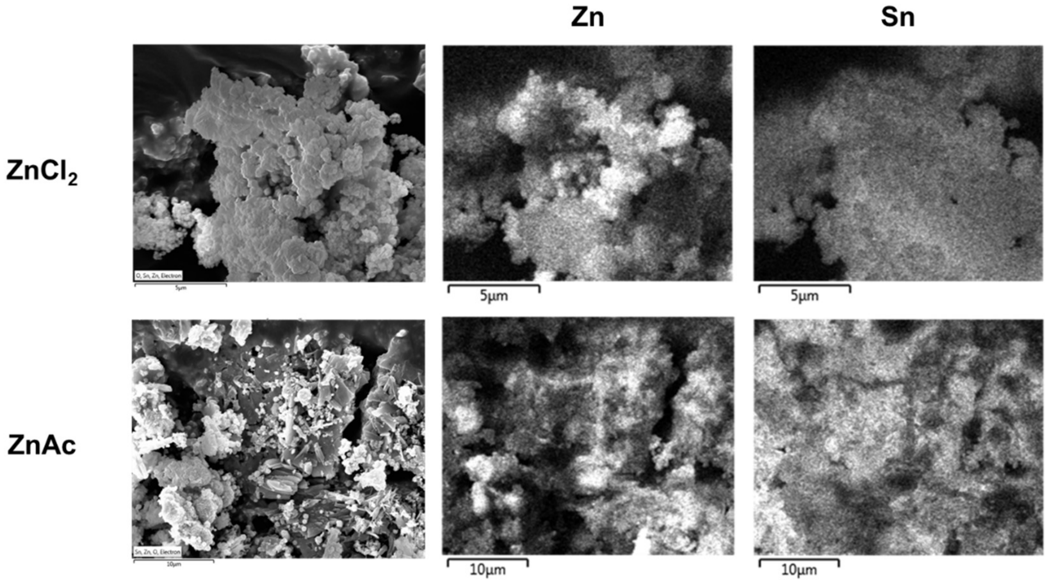

Figure S11. SEM image and EDS element maping of nanostuctures produced by synthesis using (a) $ZnCl_2$ (Zn:Sn molar ratio of 2:1) and (b) ZnAc (Zn:Sn molar ratio of 1:1), both at 200 °C for 2 h and a volume of 15 ml.

4. References

1. Zeng, J.; Xin, M.; Li, K.; Wang, H.; Yan, H.; Zhang, W. Transformation process and photocatalytic activities of hydrothermally synthesized Zn2SnO4 nanocrystals. *J. Phys. Chem. C* **2008**, *112*, 4159–4167.

2. Montenegro, D.N.; Hortelano, V.; Martínez, O.; Martínez-Tomas, M.C.; Sallet, V.; Muñoz-Sanjosé, V.; Jiménez, J. Non-radiative recombination centres in catalyst-free ZnO nanorods grown by atmospheric-metal organic chemical vapour deposition. *J. Phys.*

D. Appl. Phys. **2013**, *46*, 235302.

3. Bora, T.; Al-Hinai, M.H.; Al-Hinai, A.T.; Dutta, J. Phase Transformation of Metastable ZnSnO 3 Upon Thermal Decomposition by In-Situ Temperature-Dependent Raman Spectroscopy. *J. Am. Ceram. Soc.* **2015**, *98*, 4044–4049.

4. Choi, K.H.; Siddiqui, G.U.; Yang, B.; Mustafa, M. Synthesis of ZnSnO3 nanocubes and thin film fabrication of (ZnSnO3/PMMA) composite through electrospray deposition. *J. Mater. Sci. Mater. Electron.* **2015**, *26*, 5690–5696.
